# Digital Instruments for Reporting of Gastrointestinal Symptoms in Clinical Trials: Comparison of End-of-Day Diaries Versus the Experience Sampling Method

**DOI:** 10.2196/31678

**Published:** 2021-11-24

**Authors:** Abraham B Beckers, Johanna T W Snijkers, Zsa Zsa R M Weerts, Lisa Vork, Tim Klaassen, Fabienne G M Smeets, Ad A M Masclee, Daniel Keszthelyi

**Affiliations:** 1 Division of Gastroenterology-Hepatology Department of Internal Medicine Maastricht University Medical Center Maastricht Netherlands

**Keywords:** irritable bowel syndrome, functional dyspepsia, digital diary, experience sampling method, smartphone app, mobile phone application, mHealth, eHealth, compliance, patient-reported outcome measures

## Abstract

**Background:**

Questionnaires are necessary tools for assessing symptoms of disorders of the brain-gut interaction in clinical trials. We previously reported on the excellent adherence to a smartphone app used as symptom diary in a randomized clinical trial on irritable bowel syndrome (IBS). Other sampling methods, such as the experience sampling method (ESM), are better equipped to measure symptom variability over time and provide useful information regarding possible symptom triggers, and they are free of ecological and recall bias. The high frequency of measurements, however, could limit the feasibility of ESM in clinical trials.

**Objective:**

This study aimed to compare the adherence rates of a smartphone-based end-of-day diary and ESM for symptom assessment in IBS and functional dyspepsia (FD).

**Methods:**

Data from 4 separate studies were included. Patients with IBS participated in a randomized controlled trial, which involved a smartphone end-of-day diary for a 2+8-week (pretreatment + treatment) period, and an observational study in which patients completed ESM assessments using a smartphone app for 1 week. Patients with FD participated in a randomized controlled trial, which involved a smartphone end-of-day diary for a 2+12-week (pretreatment + treatment) period, and an observational study in which patients completed ESM assessments using a smartphone app for 1 week. Adherence rates were compared between these 2 symptom sampling methods.

**Results:**

In total, 25 patients with IBS and 15 patients with FD were included. Overall adherence rates for the end-of-day diaries were significantly higher than those for ESM (IBS: 92.7% vs 69.8%, FD: 90.1% vs 61.4%, respectively).

**Conclusions:**

This study demonstrates excellent adherence rates for smartphone app–based end-of-day diaries as used in 2 separate clinical trials. Overall adherence rates for ESM were significantly lower, rendering it more suitable for intermittent sampling periods rather than continuous sampling during longer clinical trials.

## Introduction

Disorders of brain-gut interaction (DBGI) are highly prevalent disorders, with a recent multinational survey study indicating that over 40% of the world’s population has at least 1 DBGI [1]. Among the most common DBGI are irritable bowel syndrome (IBS) and functional dyspepsia (FD), which are characterized by lower and upper gastrointestinal symptoms, respectively, including abdominal pain, fullness, bloating, constipation, and diarrhea. Per definition, the diagnosis of these disorders is symptom-based, according to the Rome IV criteria [2]. By extension, the evaluation of treatment responses in clinical trials on DBGI relies completely on patient-reported outcome measures (PROMs). It is therefore of utmost importance that clinical trials use symptom sampling methods that are able to produce an accurate representation of the symptomatology as experienced by the patient. As such, paper symptom diaries have been scrutinized as they are prone to fake adherence, as subjects can fake or backfill written answers outside of the proposed time window to forge good adherence [3]. Thereby, the use of paper retrospective diaries introduces ecological and recall bias.

End-of-day symptom diaries are currently recommended by drug regulatory authorities to assess treatment response in IBS [4,5]. The widespread dissemination of the smartphone during the previous 2 decades creates possibilities for developing more advanced symptom sampling methods. We recently reported a digital end-of-day symptom diary using a smartphone app in a randomized controlled trial (RCT) on IBS [6]. We observed a very high adherence of almost 88% for the diary smartphone app during a treatment period of 8 weeks. End-of-day diaries in any form, however, are not free of the abovementioned ecological and recall biases owing to their retrospective nature. These limitations are currently best overcome by the experience sampling method (ESM), which employs random and repeated assessments at multiple time points across momentary states in daily life and thereby provides a detailed overview of symptoms experienced during the day. Previously developed ESM instruments for FD and IBS use a measurement frequency of 10 times a day [7,8]. It should be noted that the high frequency of this sampling method might raise concerns regarding adherence during clinical trials with a duration of several weeks or longer. In our previous IBS trial using the end-of-day diary, adherence declined over time. Logging fatigue is considered the underlying cause of this decline in adherence. It could be hypothesized that this mechanism impairs adherence even more in methods with a higher sampling frequency, such as ESM. Hence, although ESM proved to be the method with more accurate real-life representation of daily symptoms, its feasibility in longer-duration clinical trials is still unknown. To draw conclusions regarding this question, a better understanding of ESM adherence and decline thereof is required.

In this exploratory study, we sought to compare adherence for end-of-day diaries used in 2 RCTs with adherence for ESM in 2 separate observational studies. We hypothesized that overall adherence would be superior with the use of the end-of-day diary, as compared to ESM. Moreover, we hypothesized that adherence would remain more stable over time for the end-of-day diary than for ESM.

## Methods

### Methods Overview

This study is based on data from 2 RCTs and 2 observational studies. For each study type, 1 study focused on IBS and 1 on FD. The RCTs used end-of-day diaries, whereas the observational studies used momentary assessments (ESM). The Rome IV criteria were used as inclusion criteria in each study in accordance with the disorder being investigated. All 4 studies had been approved by the Maastricht University Medical Center+ (MUMC+) ethics committee. All study procedures were performed in compliance with Good Clinical Practice Guidelines and in accordance with the revised Declaration of Helsinki. All subjects gave written informed consent prior to participation.

Although including data from multiple studies, all patients included in this study were required to have participated in both an RCT and an observational study (on the same disorder) to reduce variability across individuals. All patients thereby completed both end-of-day diaries during a longer period of time and ESM for a period of 1 week (not simultaneously). Details on each study are provided below, and an overview of sampling characteristics is presented in Table 1. The exact queries in each sampling method are provided in Multimedia Appendix 1.

**Table 1:** Overview of sampling specifics per study.

Sampling specifics	Randomized controlled trial on irritable bowel syndrome	Observational study on irritable bowel syndrome	Randomized controlled trial on functional dyspepsia	Observational study on functional dyspepsia
Sampling method	Digital end-of-day diary (smartphone app)	End-of-day paper diary and digital experience sampling method (smartphone app)^a^	Digital end-of-day diary (smartphone app)	Combined digital end-of-day diary and experience sampling method (smartphone app)
Sampling duration	2 weeks pretreatment + 8 weeks treatment	1 week	2 weeks pretreatment plus 12 weeks treatment	1 week
Sampling frequency	Once a day	10 times per day	Once a day	10 times per day
Sampling timeframe	Between 6 PM and 12 PM	Between 7 AM and 10 PM	Between 7 PM and 12 PM	Between 7 AM and 10 PM
Push notifications	Once at 10 PM	Randomly timed	Once at 9 PM	Randomly timed
Items, n	3^b^	32 (5 domains)	7^c^	33 (4 domains)
Estimated time investment	15-30 seconds	2-3 minutes (per assessment)	15-30 seconds	2-3 minutes (per assessment)

^a^Note that the end-of-day diaries are similar to the ones used in the corresponding randomized controlled trial. In the observational study, however, end-of-day diaries were completed on paper.

^b^In addition to items in the Bristol stool chart and adverse event and sporadic medication use queries.

^c^In addition to adverse event and sporadic medication use queries.

### RCTs Using End-of-Day Diaries

The RCT on IBS has been discussed in detail elsewhere [9]. In brief, the primary aim was to investigate the efficacy of peppermint oil, a conventional small-intestinal and a novel ileocolonic release formulation, in patients with IBS (ClinicalTrials.gov, NCT02716285). In this randomized, placebo-controlled trial, patients with IBS aged 18-75 years were included. Patients completed an end-of-day diary using a smartphone app during a pretreatment period (2 weeks) and treatment period (8 weeks), as described previously [6]. At the core, this diary consisted of 1 question regarding abdominal pain experienced each day (to be scored on an 11-point numerical scale). After completing the abdominal pain question, subjects were asked about adverse events and sporadic medication use. During the day, patients had the option to report on defecation in accordance with the Bristol stool chart [10]. Patients were instructed to register abdominal pain daily between 6 PM and 12 PM. Finally, psychological comorbidities were assessed at baseline using the General Anxiety Disorder, 7-item (GAD-7) scale and Patient Health Questionnaire, 9-item (PHQ-9).

The second RCT is an ongoing trial that investigates the efficacy of nortriptyline in patients with FD (ClinicalTrials.gov, NCT03652571). In this randomized, placebo-controlled trial, patients with FD aged 18-65 years were included. Patients completed an end-of-day diary using a smartphone app during a pretreatment period (2 weeks) and treatment period (12 weeks); the app was similar to the one used in the RCT on IBS. In the RCT on FD, however, the diary consisted of questions corresponding to the five core symptoms of FD: epigastric pain, epigastric burning, early satiety, postprandial fullness, and upper abdominal bloating [11]. In addition to these 5 questions, subjects were asked about adverse events and sporadic medication use. Patients are instructed to register symptoms daily between 7 PM and 12 PM. There was no registration of bowel movements in this trial, as an altered bowel habit is not a core symptom in FD. Finally, psychological comorbidities were assessed at baseline using the GAD-7 scale and PHQ-9.

### Observational Studies Using ESM

ESM data from patients with IBS were obtained from a validation study of a newly developed patient-reported outcome measure (based on the ESM) for the use in IBS (ClinicalTrials.gov, NCT02880722) [8]. Patients with IBS and healthy volunteers between 18 and 70 years of age were included in this study. Both groups completed an end-of-day paper diary and ESM for a period of 1 week. The ESM was incorporated in a customized smartphone app. The ESM consisted of 10 assessments randomly timed between 7:30 AM and 10:30 PM. Each assessment was preluded by an auditory signal, and the app was programmed to enable completion of the assessment within 10 minutes after the auditory signal. Subjects were instructed to complete as many assessments as possible, but to pass over questionnaires when completing was not feasible (eg, when driving). Assessments covered five different domains, as described previously [8]: physical status (eg, abdominal pain), defecation (since the previous auditory signal), psychological factors (eg, positive and negative affect), environment (eg, current location and company), and nutrition and drug use. In total, the ESM for use in IBS consisted of 32 items (per assessment).

ESM data from patients with FD were obtained from a separate validation study of a newly developed patient-reported outcome measure (based on the ESM) for the use in FD (ClinicalTrials.gov, NCT04204421) [12]. Patients with FD and healthy volunteers between 18 and 75 years of age were included in this study. Both groups completed an end-of-day diary and ESM for a period of 1 week. The diary and ESM were incorporated in the same customized smartphone app. ESM was used in a manner similar to the IBS ESM study, with 10 assessments randomly timed between 7 AM and 10 PM. Assessments in the FD ESM study covered 4 domains, which included the same as those in the IBS ESM study [7]. In total, the ESM for use in FD consisted of 33 items (per assessment).

### Statistical Analyses and Data Plots

Adherence was the primary outcome measure. For both the end-of-day diaries and ESM, overall adherence was calculated as the percentage of completed assessments throughout the study. For visualizing adherence over time, weekly adherence rates were plotted for the clinical trials (end-of-day diaries) and daily adherence rates for the observational studies (ESM). In addition, for ESM we also calculated overall adherence as the number of days on which ≥6 of the 10 assessments were completed, as described previously [8,13]. The latter can be considered more appropriate when evaluating adherence of sampling methods such as ESM, where an excess of measurements is provided to obtain sufficient data during the day [13,14].

All data were plotted using MATLAB R2018a. Linear mixed models were performed using the lme4 function in R Statistical Software (version 3.6.3, February 29, 2020) [15]. In each model (per study), adherence to the app constituted the dependent variable and time constituted the within-subject independent variable. A restricted maximum likelihood estimation method and first-order autoregressive variance-covariance matrix for the within-subject variable time fitted the data best on the basis of the lowest value of the Akaike information criterion.

Given the exploratory nature of the study, we did not perform sample size calculations.

## Results

In total, 25 patients with IBS and 15 patients with FD were included in our analysis for adherence comparison. An overview of subject characteristics is provided in Table 2. Both patients with IBS and those with FD were more frequently female (72.0% and 73.3%, respectively). For patients with FD, the time between participation in the 2 studies was significantly longer than that for patients with IBS (12.5 vs 7.6 months, respectively; *t*_26.12_=4.30; *P*<.001). All subjects in the IBS and FD studies participated in the ESM observational study after participating in the RCT.

Adherence rates for the end-of-day diary in the IBS RCT during the pretreatment period, treatment period, and total study duration (both periods combined) were 93.4%, 92.6%, and 92.7%, respectively. Overall adherence—ie, the percentage of total completed assessments—for ESM during the observational IBS study was 69.8%.

Adherence rates for the end-of-day diary in the FD RCT during the pretreatment period, treatment period, and total study duration were 92.9%, 89.7%, and 90.1%, respectively. Overall adherence for ESM during the observational FD study was 61.4%.

Of note, for trials using the ESM method, completion of ≥6 of the 10 questionnaires per day is considered as being adherent, as described previously [8,13]. This type of adherence calculation can be considered more representative for sampling methods such as ESM. When using this approach, overall adherence was 79.4% and 64.8% for the ESM IBS and ESM FD studies, respectively. Adherence in the latter was noticeably lower owing to the effect of 4 outliers (adherence<15%) in this relatively small group. Three of 4 subjects reported a specific reason for low adherence, which included (1) a technical error (subject did not receive push notifications on his/her smartphone), (2) attending the funeral of a close relative, and (3) not being able to complete most assessments during day job.

**Table 2:** Summary of patient demographic and baseline characteristics.

Characteristics			Randomized controlled trial on irritable bowel syndrome	Observational study on irritable bowel syndrome	Randomized controlled trial on functional dyspepsia	Observational study on functional dyspepsia
Subjects, n			25		15	
Time between study participations (months), mean (SD); range			7.6 (3.1); 1-17		12.5 (3.6); 7-18	
Age^a^ (years), mean (SD); range			35.9 (12.8); 22-59		41.4 (15.2); 18-64	
Females, n (%)			18 (72.0)		11 (73.3)	
**Educational level, n (%)**						
	No education		0		0	
	Low		2 (8.0%)		1 (6.7%)	
	Moderate		12 (48.0%)		5 (33.3%)	
	High		11 (44.0%)		9 (60.0%)	
Irritable bowel syndrome or functional dyspepsia subtype, n (%)			Diarrhea: 14 (56.0) Constipation: 3 (12.0) Mixed: 4 (16.0) Undefined: 4 (16.0)		Postprandial distress: 5 (33.3) Epigastric pain: 4 (26.7) Overlap: 6 (40.0)	
**Irritable bowel syndrome or functional dyspepsia severity^b^**						
	Mean score (SD)		228.8 (24.5)		83.2 (22.8)	
	Mild, n (%)		7 (28.0)		—^c^	
	Moderate, n (%)		13 (52.0)		—	
	Severe, n (%)		5 (20.0)		—	
**Psychological comorbidities^d^**						
	**Anxiety**					
		Score, mean (SD)	4.2 (2.9)		3.3 (2.8)	
		Minimal, n (%)	14 (56.0)		12 (80.0)	
		Mild, n (%)	9 (36.0)		2 (13.3)	
		Moderate, n (%)	2 (8.0)		1 (6.7)	
	**Depression**					
		Score, mean (SD)	5.0 (2.7)		4.9 (4.2)	
		Minimal, n (%)	14 (56.0)		8 (53.3)	
		Mild, n (%)	10 (40.0)		6 (40)	
		Moderate, n (%)	1 (4.0)		1 (6.7)	

^a^Age upon registering for the first study.

^b^For irritable bowel syndrome symptom severity, the Irritable Bowel Syndrome Severity Scoring System (IBS-SSS) was used. Scores were defined as follows: <175, mild; 175-300, moderate; and >300, severe. For functional dyspepsia symptom severity, the Nepean Dyspepsia Index (NDI symptom scale) was used (continuous scale only, no validated severity categories).

^c^—: not determined.

^d^For anxiety, the General Anxiety Disorder scale, 7-item, was used, and for depression, the Patient Health Questionnaire, 9-item, was used. Scores were defined as follows: ≥5, mild; ≥10, moderate; and ≥15, severe.

**Figure 1:**
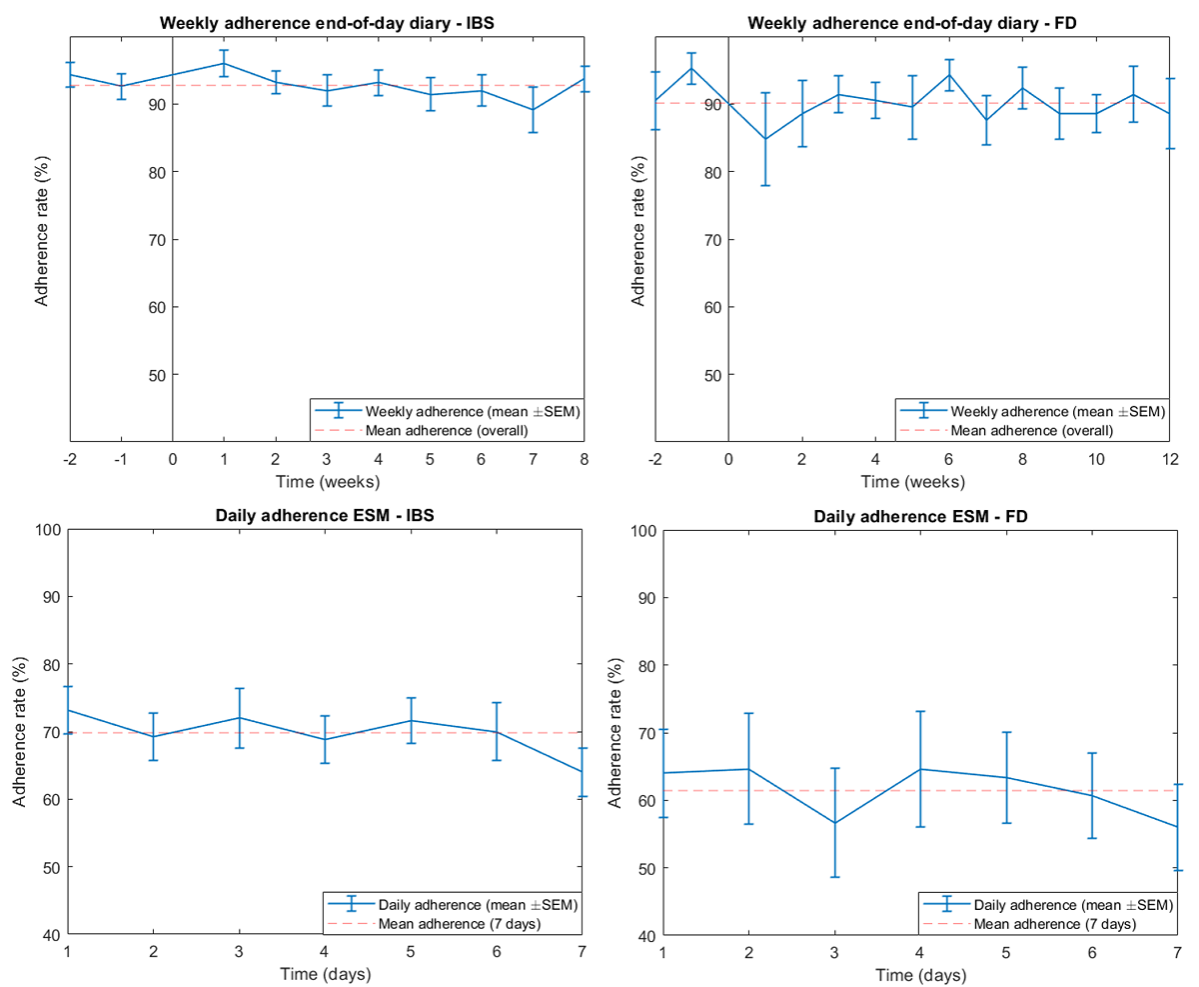
Adherence to each symptom assessment app. For end-of-day diaries, the weekly adherence is shown (ie, percentage of completed assessments for each week). For the experience sampling method, the daily adherence is shown (ie, percentage of completed assessments for each day [out of 10 measurements]). ESM: experience sampling method, FD: functional dyspepsia, IBS: irritable bowel syndrome.

Weekly adherence to the end-of-day diaries and daily adherence to the ESM app are shown in Figure 1. Linear mixed models did not demonstrate a significant decline in adherence over time in either RCT (main effect of time; IBS RCT: *F*_1,224_=2.24; *P*=.14; FD RCT: *F*_1,194_=0.87; *P*=.77). A minor decline in adherence over time can be observed in the lower panels corresponding to the ESM studies, which was not significant (linear mixed models, main effect of time; ESM IBS study: *F*_1,149_=3.41; *P*=.07; ESM FD study: *F*_1,89_=1.23; *P*=.27).

**Figure 2:**
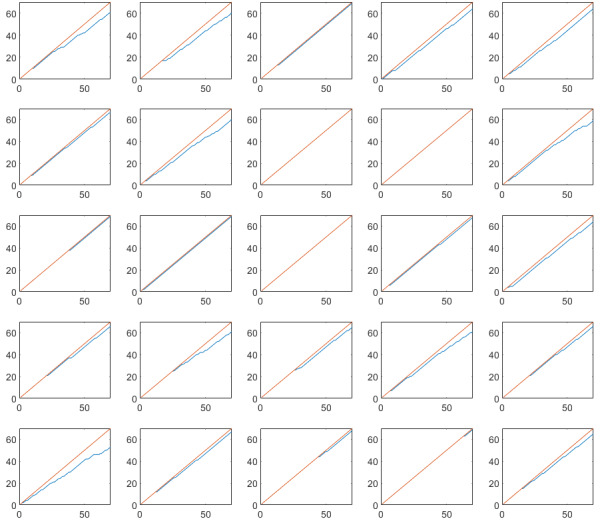
Cumulative completed diary assessments in the randomized controlled trial on irritable bowel syndrome. The red line indicates maximum number of assessments, and the blue line indicates actual number of completed assessments. Note that subplot positions in this figure correspond to the same subject in Figure 3. X-axis: assessment number, Y-axis: completed assessments.

**Figure 3:**
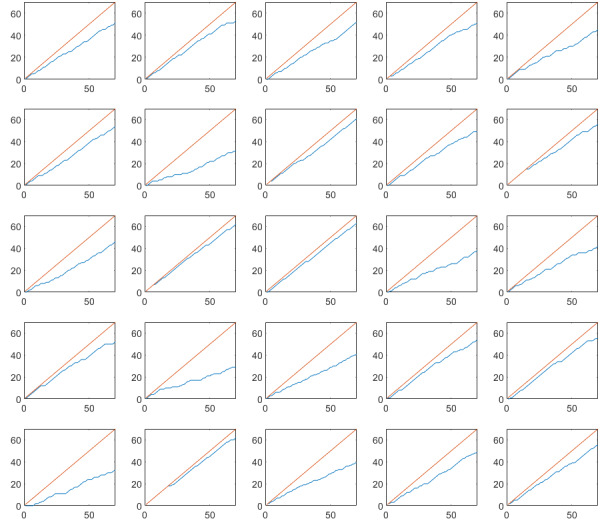
Cumulative completed experience sampling method assessments in the observational study on irritable bowel syndrome. The red line indicates maximum number of assessments, and the blue line indicates actual number of completed assessments. Note that subplot positions in this figure correspond to the same subject in Figure 2. X-axis: assessment number, Y-axis: completed assessments.

**Figure 4:**
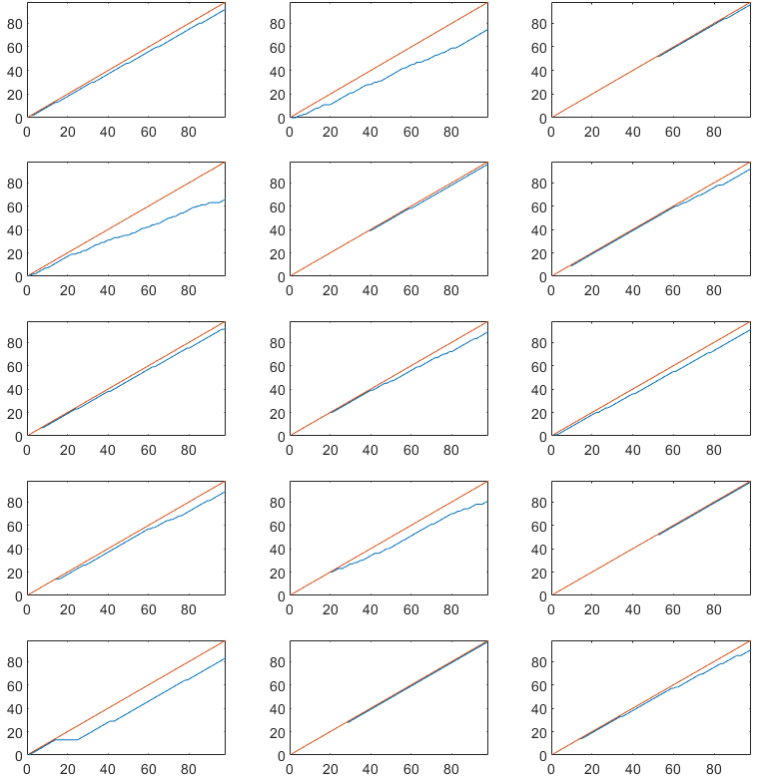
Cumulative completed diary assessments in the randomized controlled trial on functional dyspepsia. The red line indicates maximum number of assessments, and the blue line indicates actual number of completed assessments. Note that subplot positions in this figure correspond to the same subject in Figure 5. X-axis: assessment number, Y-axis: completed assessments.

**Figure 5:**
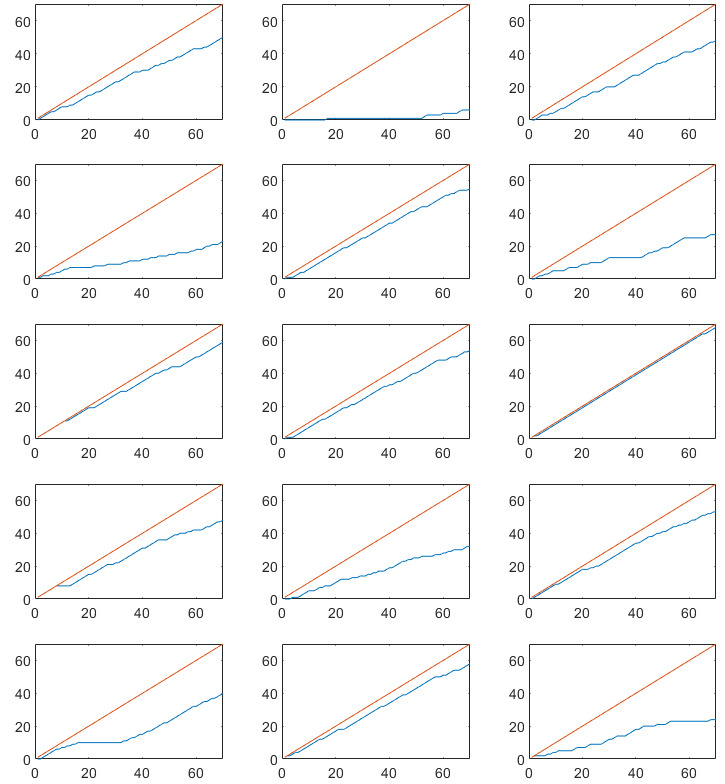
Cumulative completed experience sampling method assessments in the observational study on functional dyspepsia. The red line indicates maximum number of assessments, and the blue line indicates actual number of completed assessments. Note that subplot positions in this figure correspond to the same subject in Figure 4. X-axis: assessment number, Y-axis: completed assessments.

Cumulative completed assessments are plotted against the total number of assessments for each study and each subject in Figures 2-5. Single subject plots can be compared as subplot positions in the figures correspond to the same subject.

## Discussion

### Principal Findings

In this study, we explored adherence rates for smartphone apps used for symptom assessment in IBS and FD. In line with our previous findings in a large RCT on IBS [6], we found an excellent overall adherence of 90% for a digital end-of-day diary in an ongoing trial with patients with FD. Given that these diaries only enable the logging of symptoms experienced during the day that the diary is being filled in, fake adherence (ie, backfilling) is completely prevented. Indeed, the FDA recommends daily symptom assessment in IBS trials, which is best facilitated by the digital (smartphone) framework presented here [4]. The high overall adherence rates as observed in the FD and in the IBS RCTs confirm the feasibility of these digital diaries in clinical trials. Importantly, the RCT in FD involves 5 diary questions as opposed to a single question in the diary used in the IBS RCT. In addition, the FD RCT is 4 weeks longer in duration than the IBS RCT. It is encouraging that regardless of the added burden, adherence for the end-of-day diary in the FD RCT is still excellent.

In our previous IBS RCT where we included 189 patients, we reported a small but significant decrease in adherence for the completion of daily diaries over the study duration. Such a decrease in adherence can be referred to as logging fatigue. In the current study, we found no evidence of logging fatigue in the subset of the IBS RCT or in the FD RCT. We hypothesized that with more frequent assessments, such as with ESM, logging fatigue could become an increasing issue. Interestingly, in the current study, we found no significant decline in adherence rates over time in both ESM studies. However, since both studies were of relatively short duration (7 days), we cannot draw any conclusions on possible declining adherence rates over time when ESM is used for longer periods.

Overall adherence rates for the ESM studies were evidently lower than the end-of-day diary adherence rates, though in line with rates reported in previous studies [13,16]. Even when considering that it is generally not feasible to complete all ESM assessments and calculating ESM adherence as the number of days that ≥6 of the 10 assessments are completed, ESM adherence rates were still noticeably lower. To a large extent, this will likely be related to the nature of measurements. For the end-of-day diaries, subjects can choose a suitable moment between 6 PM (or 7 PM) and 12PM, and can do so each day as per their own schedule. For ESM, on the other hand, measurements are by their very definition timed at random moments and should be completed within 10 minutes after the assessment was announced. ESM is, therefore, likely to involve measurements at times when the subject is not able to complete the symptom assessment, especially as there will always be measurements within working hours. Furthermore, it is easy to miss a haptic or auditory signal on your cellphone. Moreover, the ESM assessments were far more extensive than the end-of-day diaries as they involved multiple domains; for example, physical, psychological, environmental, and nutritional domains. Indeed, it was demonstrated in a systematic review of studies including electronic diaries of various lengths that the extent of the diary used was negatively associated with adherence [16]. The large difference in overall adherence rates between ESM and end-of-day diaries may reflect on the higher burden of ESM. Therefore, we think that it is not feasible to use ESM continuously during a trial of several weeks, especially because adherence already tends to be lower in studies of longer duration [17]. A solution could be to use ESM intermittently (eg, 1 week in every 4 weeks), complementing the end-of-day diary. Thus, the end-of-day diary provides a strong continuous measurement framework, where ESM can be used at fixed periods to examine changes in symptom variability and symptom triggers over time (ie, during treatment), in addition to analyzing the complexity of factors contributing to symptom perception. However, the responsiveness (ie, the sensitivity to detect change over time) of ESM has not yet been evaluated.

Finally, one should appreciate the differences in acquired data when using ESM or the end-of-day diary. As already mentioned above, ESM provides more detailed information on symptoms and their possible triggers. Our preference for the end-of-day diary as a continuous measurement framework primarily relates to clinical trials, as this is also in accordance with current recommendations from regulatory authorities. It is possible that in some situations the more detailed data outweighs the drawback of the higher number of missing values. This could especially be the case in clinical practice of functional disorders, where additional information on symptom triggers is extremely valuable.

### Limitations

A limitation of the current study is the small size of the study population. This is mitigated by the within-subject nature of the study, as the obligatory participation in both an end-of-day diary and ESM study limits subject specific effects on adherence, aiding a better comparison of assessment methods. As mentioned above, the possibility of selection bias cannot be excluded, as subjects who are willing to participate in more than one study could have a very strong motivation, which may translate to unrepresentatively high adherence rates. On the other hand, the overall adherence rate of our subset of subjects from the IBS RCT was only a few percentage points higher than that of the whole group, arguing against such selection bias. Finally, since all subjects participated in the ESM studies after participation in an RCT, a carry-over treatment effect could have affected logging adherence. However, this would likely have influenced adherence during the RCT itself as well, and we observed stable adherence during both RCTs. Moreover, we previously observed no effect of GI symptoms on adherence rates. The latter also suggests that variation in duration between ESM and RCT participation is less relevant.

### Conclusions

In conclusion, we here demonstrate excellent adherence rates for smartphone app–based end-of-day diaries as well as good adherence to 2 ESM-based apps. Overall adherence rates for ESM were evidently lower, as would be expected given the nature of the methodology, but possibly also reflecting on the larger burden of this sampling method given the higher number of cues and questions to be answered. Even though we could not demonstrate a decline in response rate with ESM over a period of 7 days, it seems unfeasible to use ESM continuously in clinical trials over several weeks. Given the added value of ESM, however, researchers should consider complementing end-of-day diaries with intermittent periods of ESM.

## Appendix

Multimedia Appendix 1 Supplementary Information.

## Abbreviations

DBGI: disorders of brain-gut interaction
ESM: experience sampling method
FD: functional dyspepsia
GAD-7: General Anxiety Disorder, 7-item
IBS: irritable bowel syndrome
IBS-SSS: Irritable Bowel Syndrome Severity Scoring System
MUMC+: Maastricht University Medical Center+
NDI: Nepean Dyspepsia Index
PHQ-9: Patient Health Questionnaire, 9-item
PROM: patient-reported treatment measure
RCT: randomized controlled trial
