# Supplementary material for: Digital Instruments for Reporting of Gastrointestinal Symptoms in Clinical Trials: Comparison of End-of-Day Diaries Versus the Experience Sampling Method
Source: JMIR Form Res. 2021 Nov 24;5(11):e31678. doi: 10.2196/31678 (PMC8663435; doi:10.2196/31678)
Supplement: Multimedia Appendix 1 [file formative_v5i11e31678_app1.docx]

**Supplementary information**

Supplementary table 1. Diary questions in IBS RCT

| **Diary question** | **Symptom scale** |
| --- | --- |
| 1. How would you rate your abdominal pain today? Think about the worst abdominal pain today. | (0 = no pain, 10 = worst possible pain) |
| 1. Did you experience any side effects of the treatment? | Yes/no |
| 2a. Please specify the side effect |  |
| 2b. Please rate the severity of the side effect | (0 = not severe, 10 = very severe) |
| 2c. Please specify the duration of the side effect | …minutes, …hours |
| 1. Did you use any medication today (besides your usual medication)? | Yes/no |
| 3a. Please specify the medication |  |
| 3b. What was the dosage of the medication? | …µg, …mg, …gram, …ml, …other |
| 3c. How many times did you take the medication? | …times |
| 3d. What was the reason for taking this medication? |  |

Supplementary table 2. Diary questions in FD RCT

| **Diary question** | **Symptom scale** |
| --- | --- |
| 1. Today, I had pain in my upper abdomen. If yes, specify the worst abdominal pain today. | (0 = not at all, 10 = very severe) |
| 1. Today, I had a burning feeling in my upper abdomen. If yes, specify the worst feeling today. | (0 = not at all, 10 = very severe) |
| 1. Today, I was feeling bloated. If yes, specify the worst feeling today. | (0 = not at all, 10 = very severe) |
| 1. Today, I had a heavy feeling in my abdomen. If yes, specify the worst feeling today. | (0 = not at all, 10 = very severe) |
| 1. Today, I was not able to finish a regular sized meal. | (0 = able, 10 = not able) |
| 1. Did you experience any side effects of the treatment? | Yes/no |
| 6a. Please specify the side effect |  |
| 6b. Please rate the severity of the side effect | (0 = not severe 10 = very severe) |
| 6c. Please specify the duration of the side effect | …minutes, …hours |
| 1. Did you use any medication today (besides your usual medication)? | Yes/no |
| 7a. Please specify the medication |  |
| 7b. What was the dosage of the medication? | …µg, …mg, …gram, …ml, …other |
| 7c. How many times did you take the medication? | …times |
| 7d. What was the reason for taking this medication? |  |

Supplementary table 3. ESM questions in observational IBS study

|  | **Physical status** | **Answer scale** |
| --- | --- | --- |
| 1 | I am having abdominal pain | *0 (none) - 10 (very much)* |
| 1a | This pain is located in the following part(s) of my abdomen: | *Figure abdominal regions* |
| 2 | I am having intestinal gas | *0 (none) - 10 (very much)* |
| 2a | The intestinal gas is causing discomfort | *0 (none) - 10 (very much)* |
| 3 | I am having rumbling sounds coming from my abdomen | *0 (none) - 10 (very much)* |
| 4 | My abdomen feels bloated | *0 (none) - 10 (very much)* |
| 4a | The bloating makes me feel uncomfortable | *0 (none) - 10 (very much)* |
| 4b | The bloating is accompanied by a swollen abdomen | *0 (none) - 10 (very much)* |
| 5 | I have the urge to open my bowels | *0 (none) - 10 (very much)* |
| 6 | I am feeling sick | *0 (none) - 10 (very much)* |
| 7 | I am suffering from burping | *0 (none) - 10 (very much)* |
| 8 | I am feeling heartburn | *0 (none) - 10 (very much)* |
| 9 | I am feeling full | *0 (none) - 10 (very much)* |
| 10 | I am having palpitations | *0 (none) - 10 (very much)* |
| 11 | I am sweating | *0 (none) - 10 (very much)* |
| 12 | I am short of breath | *0 (none) - 10 (very much)* |
| 13 | I feel dizzy | *0 (none) - 10 (very much)* |
| 14 | My muscles are hurting | *0 (none) - 10 (very much)* |
| 15 | My joints are hurting | *0 (none) - 10 (very much)* |
| 16 | I feel an urge to pass urine | *0 (none) - 10 (very much)* |
|  | **Defecation** |  |
| 17 | Since the last beep, I have had the feeling that I had to open my bowels ... time(s) | *0, 1, 2, 3, 4, more than 4 times* |
| 17a | Since the last beep, I have actually opened my bowels ... time(s) | *0, 1, 2, 3, 4, more than 4 times* |
| 17b | It looked like this: ... | *Bristol Stool Score 1 - 7* |
| 17c | I had to strain | *0 (none) - 10 (very much)* |
| 17d | It feels like my bowels are not completely empty | *0 (none) - 10 (very much)* |
|  | **Mood and psychological factors** |  |
| 18 | I am feeling good | *0 (none) - 10 (very much)* |
| 19 | I am feeling low | *0 (none) - 10 (very much)* |
| 20 | I am feeling anxious | *0 (none) - 10 (very much)* |
| 21 | I am feeling irritated | *0 (none) - 10 (very much)* |
| 22 | I am feeling stressed | *0 (none) - 10 (very much)* |
| 23 | I am feeling relaxed | *0 (none) - 10 (very much)* |
| 24 | I am worried | *0 (none) - 10 (very much)* |
|  | **Context and environment** |  |
| 25 | Where am I? | *At home, at someone else's home, work/school, public place, on my way, somewhere else* |
| 26 | What am I doing (just before the beep)? | *Resting, work/school work, household work/shopping, hygiene/self-care, eating/drinking, relaxing/recreation,  sports, travelling, something else* |
| 27 | I feel (un)comfortable doing this | *-5 (extremely uncomfortable) –  5 (extremely comfortable)* |
| 28 | My symptoms are limiting my current activities | *0 (none) - 10 (very much)* |
| 29 | Who is with me? | *Partner/children, friends, housemates, colleagues, family (other than those who live in your house), acquaintances, strangers/others, no one* |
| 29a | I find this company (un)pleasant | *-5 (extremely unpleasant) - 5 (extremely pleasant)* |
|  | **Nutrition and drug use** |  |
| 30 | I have eaten ... since the last beep | *Breakfast, lunch, dinner, a snack, none of these* |
| 31 | I ate ... ago | *More than 15 min., 15–30 min.,  30 min.-1 hour, more than 1 hour* |
| 32 | Since the last beep I have used ... | *Caffeine (coffee) / theine (tea), nicotine (smoking), alcohol, drugs, medication, none of these* |
| 32a | This was following medication: | *Medication for abdominal pain, other pain relief, medication for stomach acidity, medication for nausea, medication to stop diarrhea, medication for constipation,  something else* |

Supplementary table 4. ESM questions in observational FD study

|  | **Physical status—Upper abdomen** | **Answer scale** |
| --- | --- | --- |
| 1 | I am having a full feeling in my upper abdomen | *0 (none) ‐ 10 (very much)* |
| 2 | I am having a heavy feeling in my upper abdomen | *0 (none) ‐ 10 (very much)* |
| 3 | My abdomen feels bloated | *0 (none) ‐ 10 (very much)* |
| 4 | I am having pain in my upper abdomen | *0 (none) ‐ 10 (very much)* |
| 5 | I am having a burning feeling in my upper abdomen | *0 (none) ‐ 10 (very much)* |
| 6 | I am having pain in my lower abdomen | *0 (none) ‐ 10 (very much)* |
| 7 | I feel nauseous | *0 (none) ‐ 10 (very much)* |
| 7a | Since the last beep, I have actually vomited... time(s) | *0, 1, 2, 3, 4, 5, more than 5*  *times* |
| 8 | I am suffering from bothersome burping | *0 (none) ‐ 10 (very much)* |
| 9 | I am having a burning feeling behind the breastbone | *0 (none) ‐ 10 (very much)* |
| 10 | 10 I am bringing up stomach contents into the mouth and/or nose | *0 (none) ‐ 10 (very much)* |
|  | **Nutrition, medication, and substance use** |  |
| 11 | I have eaten... since the last beep | *Breakfast, lunch, dinner, a snack, none of these* |
| 11a | I ate... ago | *Less than 15 min.,*  *15‐30 min., 30 min.‐1 hour,*  *more than 1 hour* |
| 11b | I am able to finish a normal sized meal | *0 (none) ‐ 10 (very much)* |
| 12 | Since the last beep I have used... | *Caffeine (coffee)/theine*  *(tea), nicotine (smoking),*  *alcohol, drugs, medication,*  *carbonated beverages, milk*  *products, fruit juices, none*  *of these* |
| 12a | This was medication for: | *Abdominal pain, other pain*  *relief, stomach acidity,*  *nausea, diarrhea,*  *constipation, something*  *else* |
|  | **Physical status—General complaints** |  |
| 13 | I am having palpitations | *0 (none) ‐ 10 (very much)* |
| 14 | I am sweating | *0 (none) ‐ 10 (very much)* |
| 15 | I am short of breath | *0 (none) ‐ 10 (very much)* |
| 16 | I feel dizzy | *0 (none) ‐ 10 (very much)* |
| 17 | I have pain on my chest | *0 (none) ‐ 10 (very much)* |
| 18 | I feel tired | *0 (none) ‐ 10 (very much)* |
| 19 | Since the last beep, I have had the feeling that I had to open my bowels … time(s) | *0, 1, 2, 3, 4, 5, more than 5*  *times* |
| 20 | Since the last beep, I have actually opened my bowels … time(s) | *0, 1, 2, 3, 4, 5, more than 5*  *times* |
| 20a | It looked like this: | *Bristol Stool Form Scale* |
| 20b | I had to strain | *0 (none) ‐ 10 (very much)* |
| 20c | It feels like my bowels are not completely empty | *0 (none) ‐ 10 (very much)* |
|  | **Psychological aspects** |  |
| 21 | I am feeling good | *0 (none) ‐ 10 (very much)* |
| 22 | I am feeling relaxed | *0 (none) ‐ 10 (very much)* |
| 23 | I am feeling low | *0 (none) ‐ 10 (very much)* |
| 24 | I am feeling anxious | *0 (none) ‐ 10 (very much)* |
| 25 | I am feeling irritated | *0 (none) ‐ 10 (very much)* |
| 26 | I am feeling stressed | *0 (none) ‐ 10 (very much)* |
| 27 | I am worried | *0 (none) ‐ 10 (very much)* |
|  | **Context and environment** |  |
| 28 | Where am I? | *At home, at someone else's*  *home, work/school, public*  *place, on my way,*  *somewhere else* |
| 29 | What am I doing (just before the beep)? | *Resting, work/school,*  *household work/shopping,*  *hygiene/self‐care, eating/*  *drinking, relaxing/*  *recreation, sports,*  *travelling, something else* |
| 30 | I feel (un)comfortable doing this | *-5 (extremely uncomfortable)*  *– +5 (extremely*  *comfortable)* |
| 31 | My symptoms are limiting my current activities | *0 (none) ‐ 10 (very much)* |
| 32 | Who is with me? | *Partner/children, friends,*  *housemates, colleagues,*  *family (other than those*  *who live in your house),*  *acquaintances, strangers/*  *others, no one* |
| 33 | I find this company (un)pleasant | *-5 (extremely unpleasant)*  *– +5 (extremely pleasant)* |
